# An overview of the literature on assistance dogs using text mining and topic analysis

**DOI:** 10.3389/fvets.2024.1463332

**Published:** 2024-12-11

**Authors:** Emma Bassan, Alberto Mair, Marta De Santis, Massimo Bugianelli, Enrico Loretti, Alessio Capecci, Franco Mutinelli, Laura Contalbrigo

**Affiliations:** ^1^National Reference Centre for Animal Assisted Interventions, Istituto Zooprofilattico Sperimentale delle Venezie, Legnaro, Italy; ^2^National Guide Dog School for the Blind, Scandicci, Italy; ^3^Local Healthcare Unit “Toscana Centro”, Firenze, Italy; ^4^Health, Welfare and Social Affairs Direction Regione Toscana, Firenze, Italy

**Keywords:** assistance dogs, service dogs, text mining, topic analysis, generative model, Latent Dirichlet Allocation

## Abstract

It is said that dogs are human's best friend. On occasion, dogs can be raised and trained to provide additional specific benefits to humans suffering from a range of physical or mental conditions, working as assistance dogs. In this article, we employed innovative techniques to review the vast and constantly expanding literature on the subject, which covers a multitude of aspects. The 450 articles obtained through keyword search on Scopus were initially described in terms of year of publication, geographical context and publication destination, and were subsequently analysed through automated text mining to detect the most important words contained within them. Lastly, a generative model of topic analysis (Latent Dirichlet Allocation—LDA) described the content of the collection of documents, dividing it into the appropriate number of topics. The results yielded interesting insights across all domains, demonstrating the potential of automated text mining and topic analysis as a useful tool to support the researchers in dealing with complex and time-consuming subjects' reviews, integrating the work done with traditional reviewing methods.

## 1 Introduction

The term “assistance dog” refers to a dog specifically trained to perform tasks to increase independence and mitigate the limitations of a person with a physical, mental, or cognitive disability (1, 2). These dogs perform at least one identifiable task or behaviour (excluding any form of protection, comfort, or defence) to assist a person with a disability in mitigating the impact of his/her condition. Such tasks may be context-induced or induced by the handler's voluntary verbal cueing and vary depending on the disability and the individual needs of the person. It is also essential that assistance dogs are trained to exhibit and maintain balanced behaviour in all social contexts in order to be able to access public spaces that are prohibited to most animals (3).

**Figure 1:**
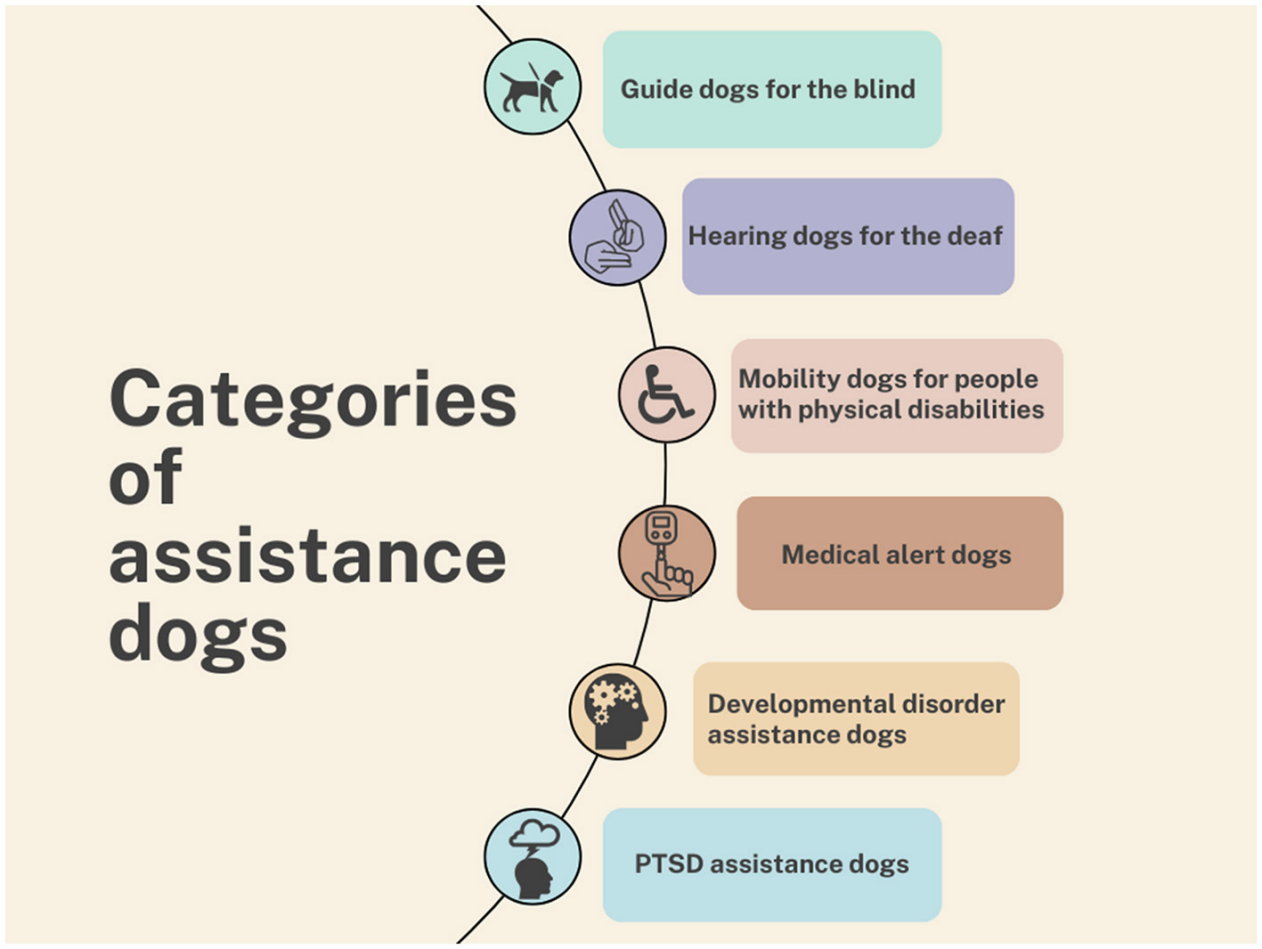
Categories of assistance dogs considered in this review.

Regarding assistance dogs, there is still no internationally agreed terminology for a universally accepted classification, and there remains some ambiguity regarding the distinction between assistance dogs and service dogs (terms that are often used interchangeably) and other categories of working or support dogs (3). This aspect has already been discussed by other authors, and several classifications have been described and proposed (3–6). For the purpose of this study, the classification proposed at the European level will be adopted (1). This classification of assistance dogs encompasses guide dogs for blind and visually impaired persons, hearing dogs for deaf persons, mobility assistance dogs for people with physical disabilities, assistance dogs for people suffering from post-traumatic stress disorder (PTSD), medical alert dogs, autism, and developmental disorder assistance dogs. In contrast, the following categories are excluded from the scope of assistance dogs: assisted intervention dogs, facility dogs, emotional support dogs, skilled companion dogs, and all other categories of working dogs—such as those in the armed forces and the police, search dogs on the surface or in rubble, detection dogs, and so forth (1). Figure 1 illustrates the aforementioned categories, which are collectively referred to as “assistance dogs” in accordance with the European-level definition cited in this article (1).

This categorisation is primarily based on the handling and objectives of dog's employment. Assistance dogs provide constant support in the life of the person with a disability, whether the dog is directly cared for by the patient in an ongoing one-to-one relationship or by another support person, such as a parent or spouse (1). In contrast, dogs involved in canine assisted interventions (CAI) can also provide support to people with disabilities, but they do not accompany the individual with a disability in daily life and do not receive targeted training to support a specific patient. Moreover, they do not reside with the individual with disability, but are entrusted to a professional handler. These dogs work in specific facilities or at the patient's home, where they carry out animal-assisted therapy (AAT), animal-assisted education (AAE), or animal-assisted activity (AAA) sessions (2, 7). Additionally, the term “facility dog” is used to describe dogs that have undergone training to work in certain facilities, such as hospitals or schools, or in specific contexts, such as legal environments. The animal's residence can vary, being either at the facility or in an outdoor environment (3). Finally, an emotional support dog provides comfort and companionship to a person suffering from a mental illness, but he/she is not trained to perform specific tasks. Usually, these animals are indistinguishable from companion animals, with the exception that the owner must have a disability and experience some alleviation of symptoms thanks to the animal (3).

The field of assistance dogs has witnessed a surge in recent years, accompanied by an intensified drive towards global standardisation. Concurrently, the number of academic publications on the subject has grown, reflecting the growing interest of the scientific community (8–10). In particular, a scoping review of interdisciplinary research concerning the categories and areas of employment of service dogs, performed on scientific literature until 2019, inspired us to perform an updated review of the current literature regarding assistance dogs (11). To explore the topic, we employed an innovative research technique based on machine learning, text mining, and topic analysis, which has been previously applied to the analysis of scientific literature on other topics (12–17). Text mining and topic analysis technologies allow knowledge to be extracted from large amounts of unstructured data by analysing massive amounts of textual data, such as is typically required in a literature review. The process works by exploring the relevant rules that exist in large amounts of text, revealing hidden links within words and clustering topics to identify research trends. In the case of scientific literature reviews, it offers the possibility of analysing a large number of articles, which would be very time-consuming to analyze using traditional review methods, and limiting some of the possible biases in the process of synthesis (18, 19).

Therefore, the objective of this study was to utilise text mining and topic analysis techniques to examine interdisciplinary scientific literature pertaining to assistance dogs, with a view to identifying trends in research on the topic.

## 2 Methods

### 2.1 Literature search, selection process, and information on the articles included

A preliminary search was conducted on the Scopus platform, the bibliometric database of Elsevier, to explore the topic of assistance dogs. The search string was composed of keywords normally associated with each category of assistance dogs (as identified in Figure 1), including terms such as “assist^*^ dog^*^,” “service dog^*^,” “guid^*^ dog^*^,” “hear^*^ dog^*^,” “mobility assist^*^ dog^*^,” “alert^*^ dog^*^,” “medical alert^*^ assist^*^ dog^*^,” “medical respon^*^ dog^*^,” “medical respon^*^ assist^*^ dog^*^,” “seizure-alert dog^*^,” “diabet^*^ alert^*^ dog^*^,” “autis^*^ assist^*^ dog^*^,” “PTSD assist^*^ dog^*^,” and “development^*^ disorder assist^*^ dog^*^.”

According to the elimination of redundant keywords, the search string was refined and the definitive search was conducted on Scopus on 8 February 2024. The search was conducted on titles, keywords, and abstracts of the papers. This yielded a total of 1,268 scientific papers. Subsequently, the document type was limited to include only articles, resulting in 819 articles. Other document types, such as conference papers, reviews, book chapters, notes, letters, editorials, books, conference reviews, errata, short surveys, and retracted papers were excluded. This was done because subsequent analyses will only be conducted on the abstracts of the included records. Abstracts may not always be present or may be presented differently in other types of documents than original research articles. Reviews were also excluded to prevent redundancy of information and content in text analysis. Additionally, the language was restricted to English, resulting in 756 articles. There were no limitations placed on the publication date. Therefore, the search string used in this final search, including language and document type limitations, is as follows: TITLE-ABS-KEY [(“assist^*^ dog^*^” OR “service dog^*^” OR “guid^*^ dog^*^” OR “hear^*^ dog^*^” OR “alert^*^ dog^*^” OR “medical respon^*^ dog^*^” OR “seizure-alert dog^*^” OR “diabet^*^ alert^*^ dog^*^”)] AND [LIMIT-TO (LANGUAGE, “English”)] AND [LIMIT-TO (DOCTYPE, “ar”)].

The 756 records were downloaded and organised in a Microsoft Excel sheet, where papers without an abstract (53) were automatically excluded. The remaining 703 titles and abstracts were screened, excluding duplicates and papers with incorrect document types or language. A further manual selection was conducted by one of the researchers to identify titles and abstracts that were not relevant to assistance dogs, which were then discussed with the research team. The approved papers underwent a second screening by two additional reviewers. Successively, information was extracted from Scopus on the characteristics of the included articles, specifically the year and country of publication, the journal name, and the subject area. This information was then synthesised numerically and narratively and presented using figures, tables, or graphs (paragraph 3.1).

### 2.2 Text mining

An Excel file was created from the selected articles, with each abstract assigned a unique numerical identifier. The texts of the included abstracts have been corrected for typographical errors and instances of semi-duplicate words that were written in American English, while the language used to analyze the texts was British English. The words of the abstract were first tokenized by splitting the text documents into single-word units. From the list of tokenized words, a list of English stop words (i.e., the most common, ubiquitous words of the English language) has been excluded. Another list containing common words resulting from our list of keywords (“dog,” “dogs,” “animal,” “animals,” “service,” “assistance,” “guide”) and other words typical of scientific articles in general (“support,” “result,” “results,” “research,” “people,” “author,” “authors,” “study,” “studies”) was excluded from the analysis, to avoid these common words being mistaken for crucial words. The words “n” and “p” were also included in the stop words list because of their ubiquitous presence in scientific papers, indicating respectively the numerousness of the experimental sample and the probability to have found a particular set of observations if the null hypothesis were true (*p*-value). Lastly, all the instances of the term “post-traumatic stress disorder” have been replaced by the acronym “PTSD” to avoid the distortion in the correlation matrices, used to analyze the results of the text mining method caused by the constant appearance of four words in a row. The remaining list of words underwent a stemming procedure, reducing the words to their roots, to avoid double-counting plurals or derivations of a word. It can be inferred from the results section that the stemming process led to the differentiation between the diverse functions of the same root, resulting in the generation of distinct elements, contingent upon the grammatical category of the root: whether it functions as a verb, adjective, or noun.

Two statistics were calculated to understand the relevance of each word in our set of abstracts: term frequency (20) and inverse document frequency (21). Term Frequency (TF) is the ratio of each word count to the total number of words in all the documents. Inverse Document Frequency (IDF) is calculated by log scaling the ratio of the number of documents to the number of documents that contain the target word. IDF is therefore a statistic indicating the inverse frequency of a word over a range of documents. The highest IDF values are assigned to the words that appear in only one document. The combination of these two statistics through simple multiplication returns the TF_IDF index (22), which determines the relevance of a word by balancing its overall frequency (TF) with its specificity (IDF). A word is less important than an equally frequent word that appears in fewer documents because that word must be more widespread and thus more important in the few documents where it appears. The 25 words with highest TF_IDF index were reported with a histogram. The TF and IDF values were also reported to better illustrate the relevance components of the words. Additionally, the words that appeared more frequently in the same documents as the 25 most relevant words (calculated correlation ≥ 0.3) were reported. The correlation represents an association between the 25 most relevant words and the rest of terms of the set on the basis of co-occurrence in the same texts. This text mining analysis has been performed in an R environment (23) using functions from the packages “tidytext” (24), “tm” (25), “SnowballC” (26), and “widyr” (27).

### 2.3 Topic analysis

The topic analysis (TA) was performed using Latent Dirichlet Allocation (LDA) (28), a generative statistical modelling technique able to extract topics from collections of texts. Each document is divided into a set of topics, each comprising a set of terms that could share a common theme. The model infers the collection of topics across the different records and the possible distribution of terms that belong to them. This topic analysis has been performed and presented in graphic form in an R environment using functions from the packages “textstem” (29), “topicmodels” (30), and “tidytext” (24).

Prior to the commencement of the TA, the number of topics into which the corpus had to be split was determined. In fact, the LDA function used to perform the automatic extraction of the topics requires an *a-priori* definition of the number of topics to be fitted. However, because the “ideal” number is generally unknown, we had to use a computing method to estimate it. The “ldatuning” package (31) allows for an estimation of this parameter based on the comparison of four different methods (32–35). Two of them (32, 33) aim at the minimisation of the divergence values of the matrices calculated by the models, meaning the differences internal to the set of topics; while the other two (34, 35) aim at the maximisation of the differences between topics. The results of the crossing between the results of the four methods, visualised with the “FindTopicsNumber_plot” function of the “topicmodels” package (30), should return a correct estimation of a representative number of topics to be fitted by the model.

## 3 Results

### 3.1 Number and characteristics of included articles

**Figure 2:**
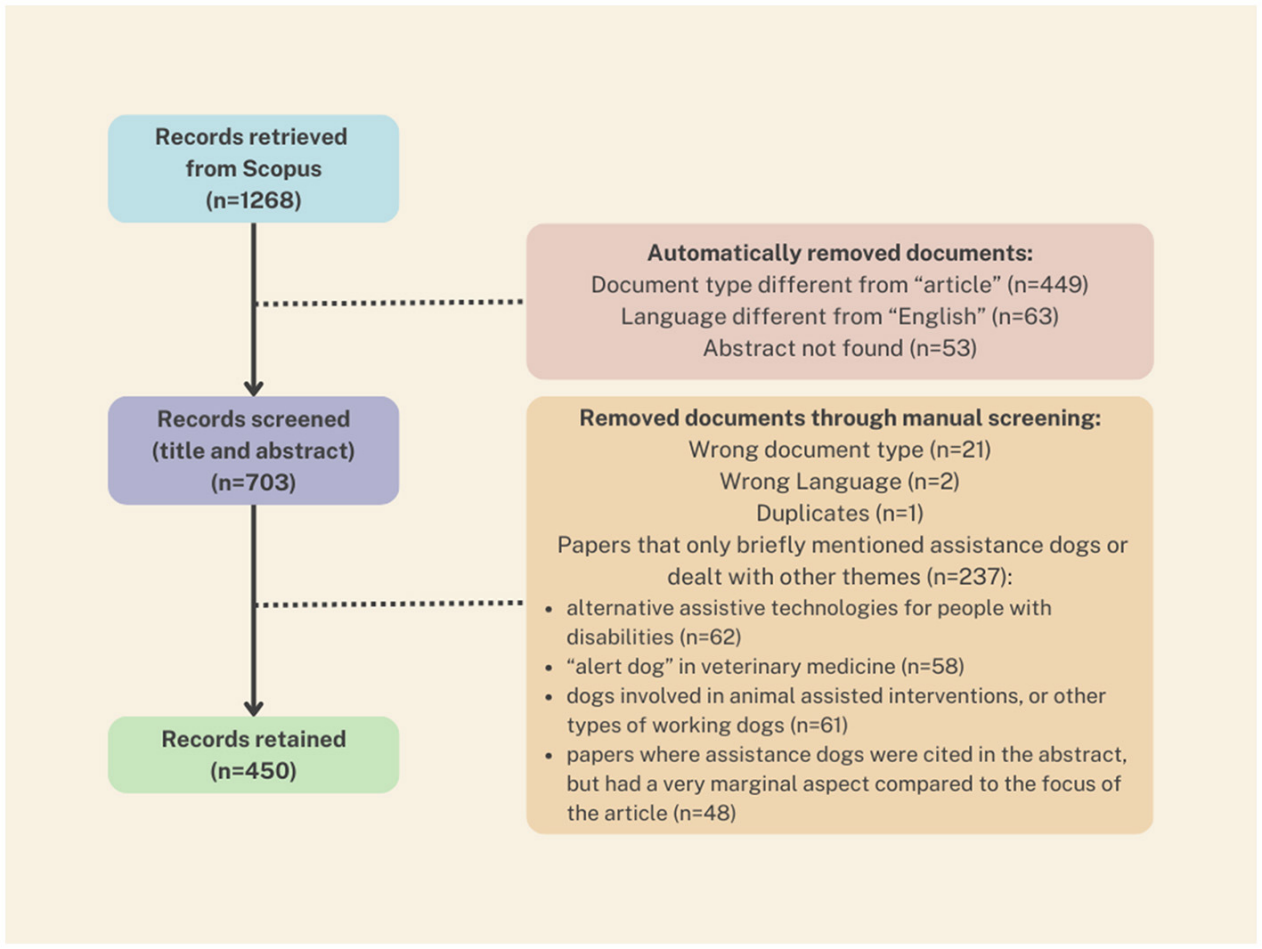
Flow chart of the screening process of scientific literature concerning assistance dogs.

The selection process yielded 450 articles deemed suitable for text mining analysis. Figure 2 displays the details of the selection process for the retrieved articles, performed by screening titles and abstracts.

**Figure 3:**
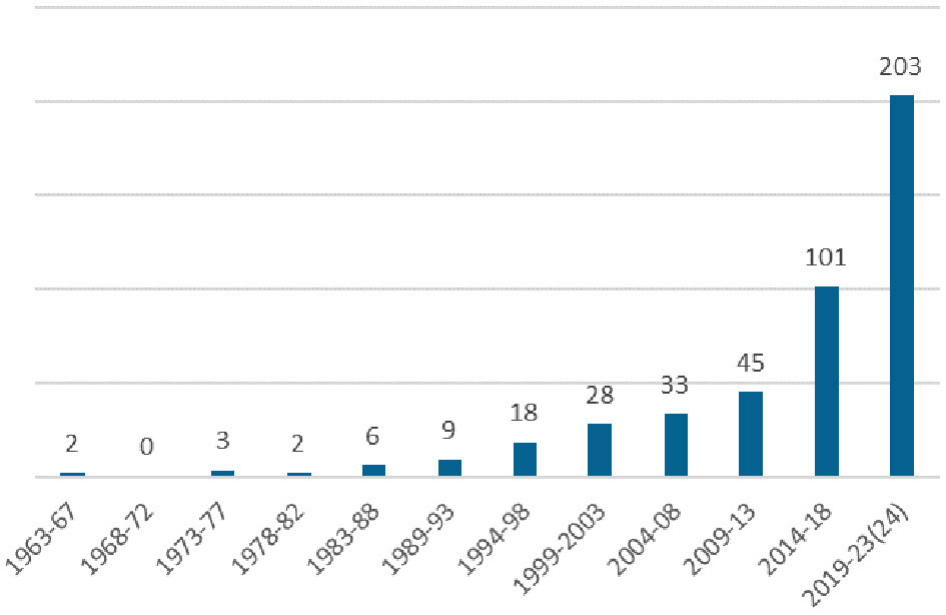
Distribution of studies published by 5-year intervals (*n* = 450). The last interval (2019–2023) includes the first 2 months of 2024, until the date of search (8th of February 2024).

**Figure 4:**
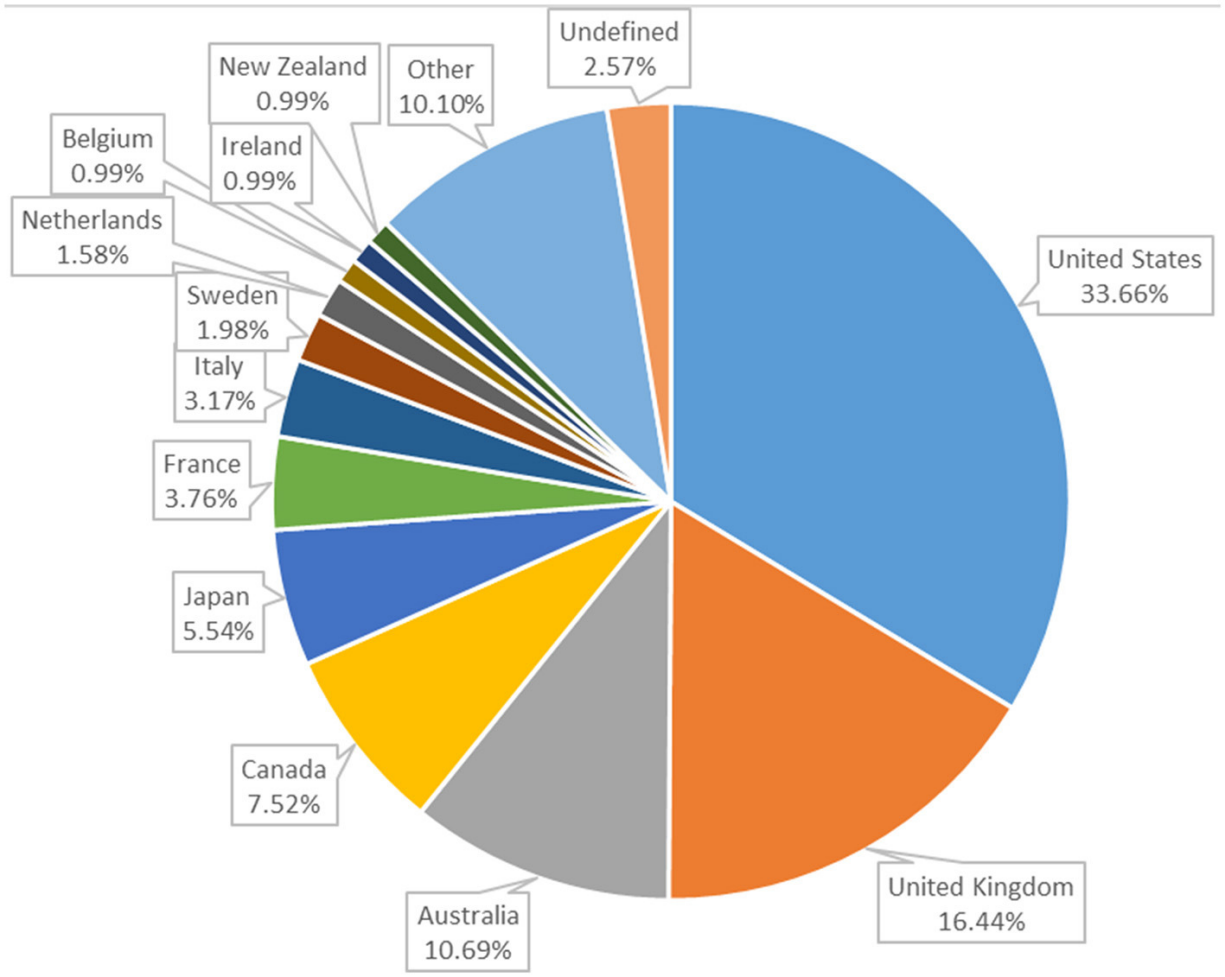
Distribution of studies (*n* = 450) by country. It should be noted that some studies are included in more than one country. The category other (*n* = 51) includes Austria, Denmark, Germany, Israel, Estonia, Finland, Hungary, Norway, Spain, Brazil, China, Croatia, Mexico, South Korea, Argentina, India, Luxembourg, Morocco, Palau, Singapore, South Africa, Switzerland, Trinidad and Tobago, and Ukraine.

Considering the year of publication of the included articles, the number of articles published in the last 10 years (304) accounts for almost 70% of the total number of articles included, the first of which dates back to 1963 (Figure 3). The countries of publication, derived from the authors' institutional affiliations, are presented in Figure 4. The majority of papers (253) were attributed to the United States (170) and the United Kingdom (83).

**Figure 5:**
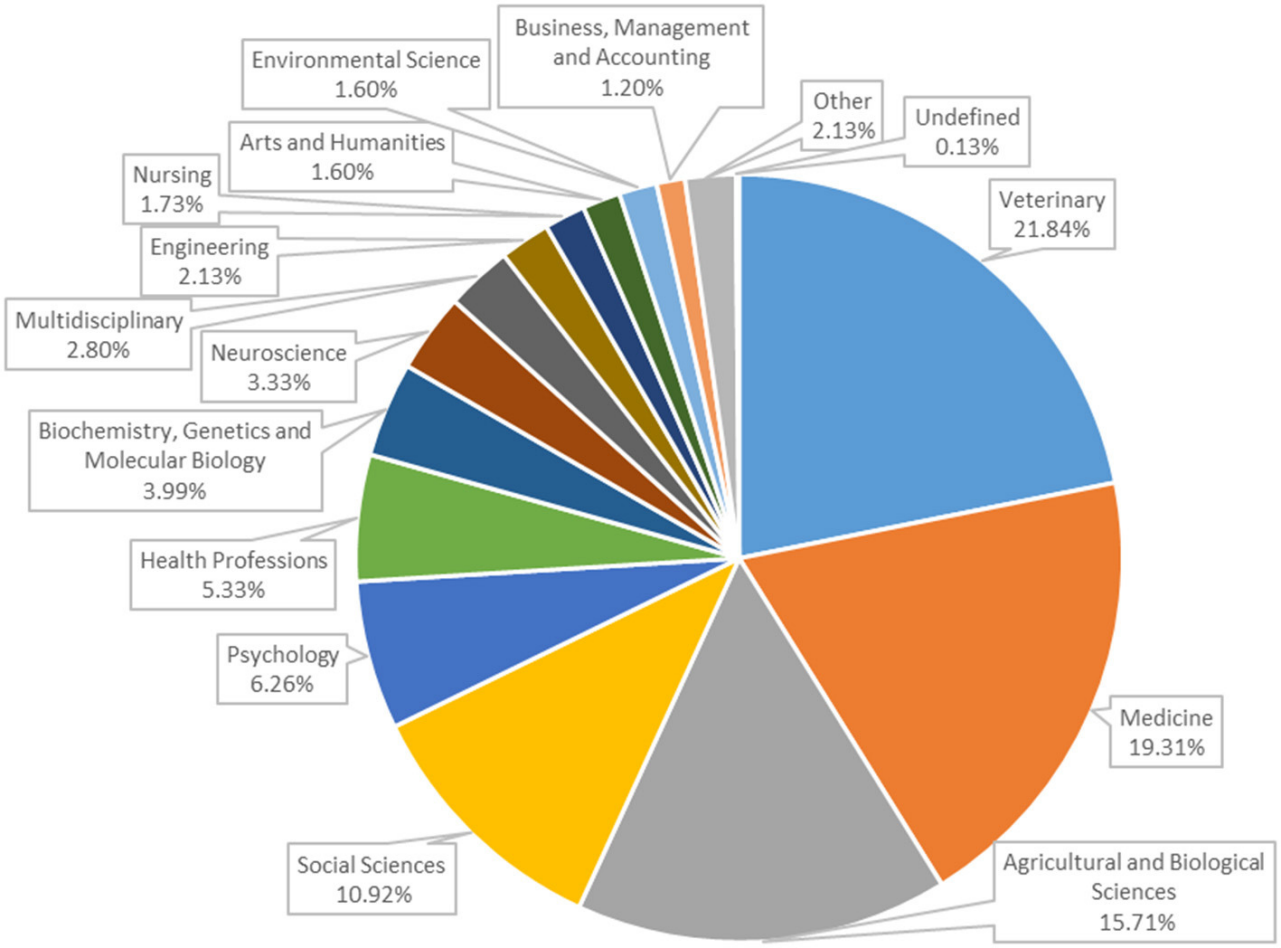
Distribution of studies (*n* = 450) by subject area. Some studies are included in more than one subject. The category other (*n* = 16) includes Chemical Engineering, Chemistry, Computer Science, Economics, Econometrics and Finance, Physics and Astronomy, Energy, Immunology, and Microbiology.

The total number of different journals in which the articles were published was 210. The seven most prolific publishers, as indicated by the number of included articles published in their journals, are presented in Table 1. Figure 5 illustrates the distribution of articles by subject area, as classified by Scopus. The subject areas of veterinary medicine and medicine accounted for 21.8 and 19.3% of the totality of subject areas, respectively.

**Table 1:** List of journals publishing 10 or more articles on the topic of interest of this review.

**Journal title**	***n* (%)**
Applied Animal Behaviour Science	28 (6.2%)
Animals	24 (5.3%)
Frontiers in Veterinary Science	18 (4%)
Anthrozoos	17 (3.7%)
Journal of Veterinary Behaviour: Clinical Applications and Research	15 (3.3%)
PLoS ONE	15 (3.3%)
Disability and Rehabilitation: Assistive Technology	10 (2.2%)

### 3.2 Text mining

**Figure 6:**
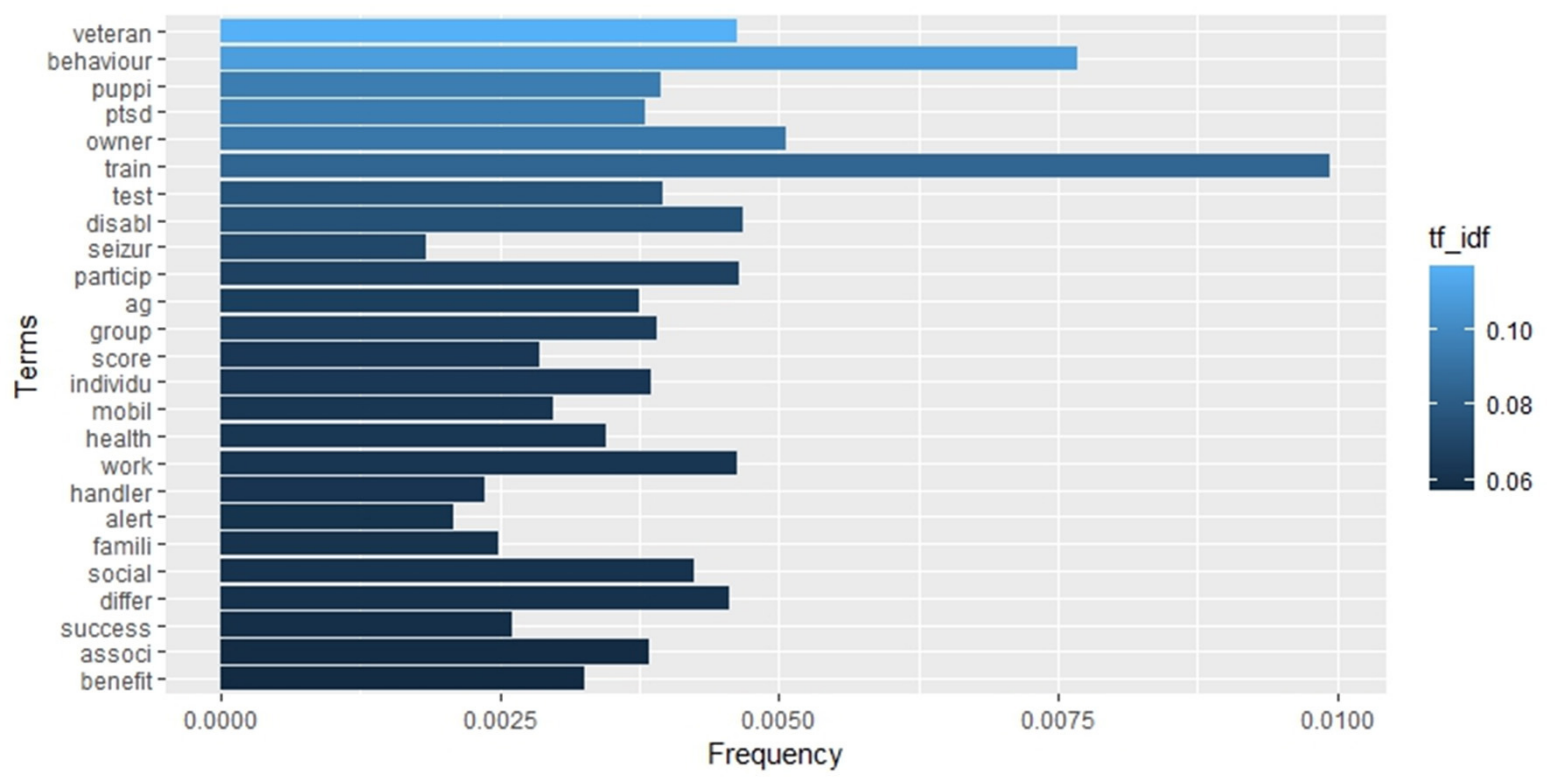
Barplot representing the frequency rate of the 25 most relevant (TF_IDF) terms in the whole collection of documents.

**Figure 7:**
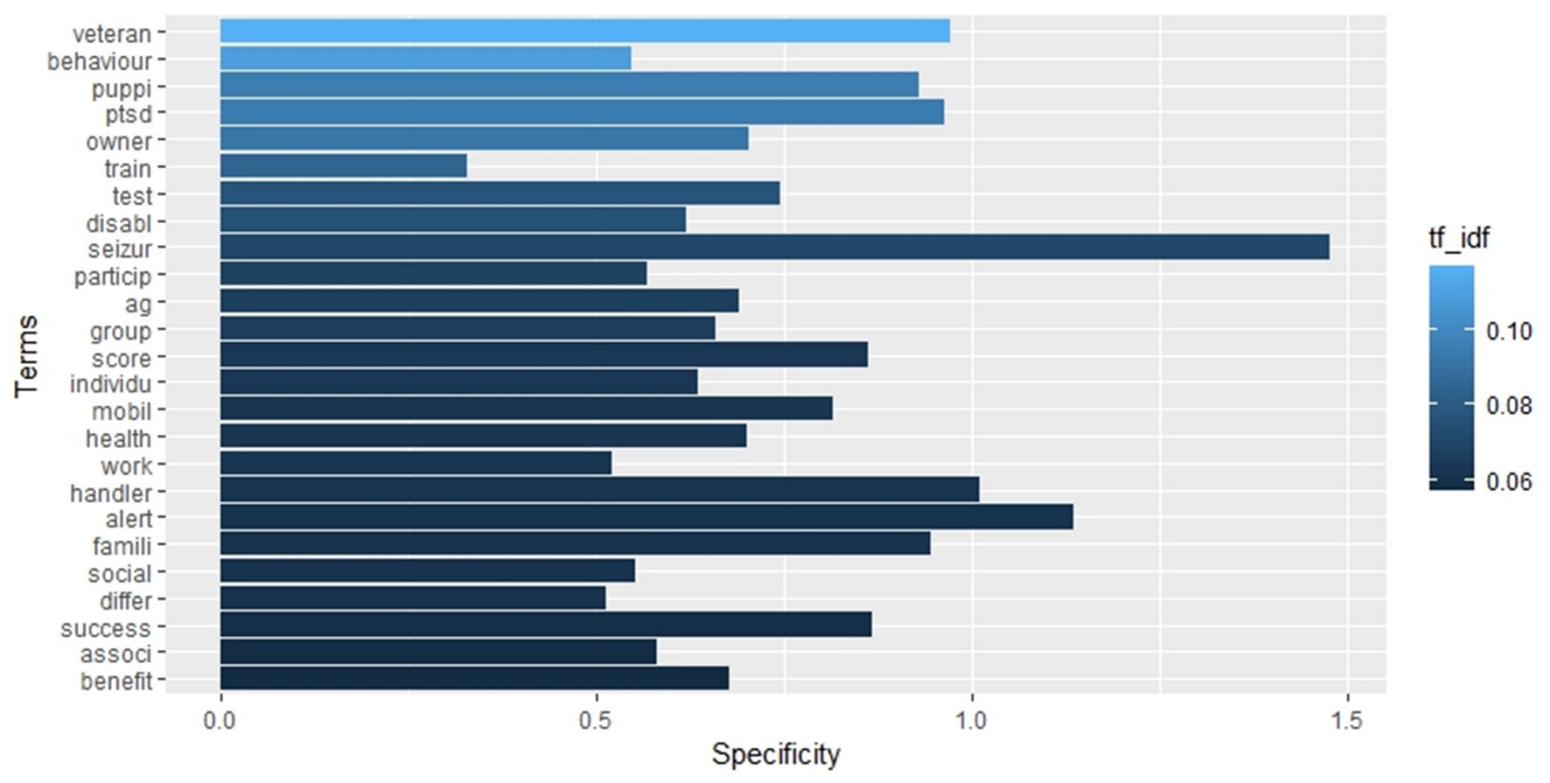
Barplot representing the inverse document frequency of the 25 most relevant (TF_IDF) terms in the whole collection of documents.

**Figure 8:**
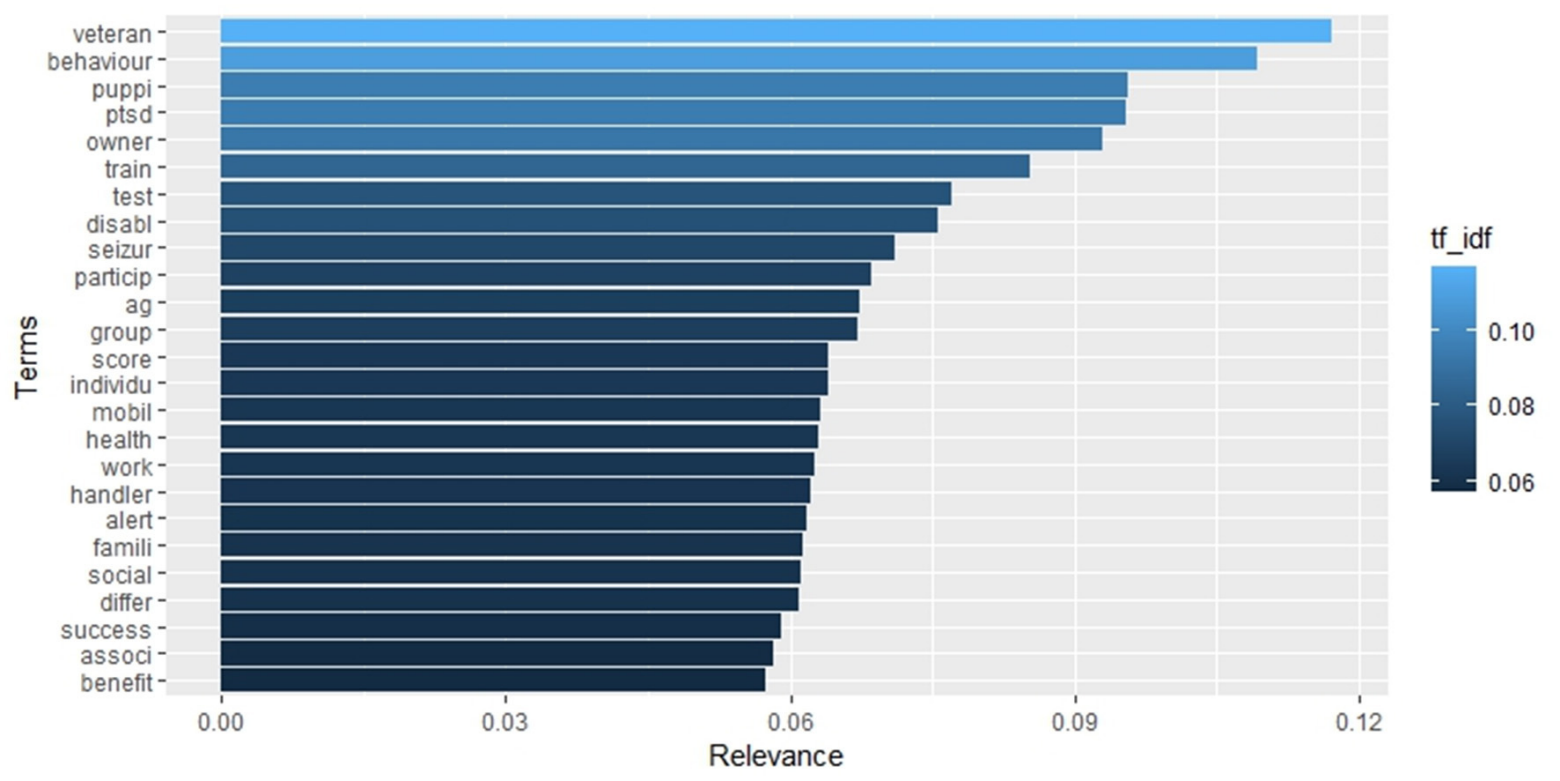
Barplot representing the list of the 25 most relevant (TF_IDF) terms in the whole collection of documents.

The term frequency analysis is illustrated in the histogram in Figure 6, which displays the 25 most frequent words. In contrast, Figures 7, 8 depict the 25 most specific and the 25 most relevant (TF_IDF ≥ 0.057) words of this set, respectively. As previously stated in the methodology section, the specificity of a word is calculated through IDF, with the highest IDF values assigned to those that appear in only one document. In contrast, the relevance of a word is calculated through TF_IDF, with the overall frequency (TF) of a word balanced against its specificity (IDF). It is important to note that there may be discrepancies between the three histograms. These discrepancies can be observed in Figures 6, 7, where the colour gradation and the order reflect the TD_IDF values relative to each word, while the length of the bars reflects the TF and IDF index, respectively.

Table 2 lists the strongest associations (correlation ≥ 0.3) between the 25 most relevant words (Figure 8) and the rest of terms of the set. Ten words on 25, namely “associ,” “benefit,” “differ,” “handler,” “individu,” “owner,” “social,” “test,” “train,” and “work,” showed no significant correlation with other words.

**Table 2:** Associations between the most relevant words (TD_IDF ≥ 0.057) and the other words present in the corpus of records.

**Top 25 relevant words**	**Words associated (correlation ≥0.3)**
Veteran	PTSD (0.85), symptom (0.6), military (0.59), psychiatry (0.5), complementari (0.5), sleep (0.43), post (0.4), civilian (0.36), treatment (0.35), intervent (0.34), waitlist (0.33)
Behaviour	Trait (0.30)
Puppi	Raiser (0.61), rais (0.4), earli (0.39), old (0.33), predict (0.31), volunt (0.3)
PTSD (Post-traumatic stress disorder)	Veteran (0.85), symptom (0.64), military (0.58), complementari (0.49), psychiatry (0.46), sleep (0.46), post (0.50), treatment (0.42), intervent (0.38), adjunct (0.38), waitlist (0.36), mental (0.34), suicid (0.33), particip (0.33), trauma (0.33), depress (0.32), size (0.31), particip (0.31)
Disabl	American (0.34)
Seizur	Epilepsi (0.66), epilept (0.57), spontan (0.48), alert (0.47), warn (0.44), refin (0.38), consent (0.36), denver (0.36), imprint (0.36), clonic (0.36), tonic (0.36), ictal (0.36), admiss (0.36), overt (0.36), preced (0.33), recogn (0.31), onset (0.31)
Particip	PTSD (0.33)
Ag	Month (0.31), gender (0.3), sex (0.3)
Group	Control (0.31)
Score	Scale (0.35)
Mobil	Impair (0.38), travel (0.33), cane (0.31)
Health	Mental (0.31)
Alert	Diabet (0.49), seizur (0.47), hypoglycaem (0.45), hypo (0.41), hypoglycaemia (0.41), glucose (0.4), dad (0.38), epilepsi (0.38), monitor (0.34), hyperglycaem (0.34), detect (0.31), episode (0.31), spontan (0.31)
Famili	Autism (0.43), children (0.42), parent (0.35), autist (0.32), member (0.31), child (0.31)
Success	Failure (0.36), releas (0.34), successfully (0.33)

### 3.3 Topic analysis

**Figure 9:**
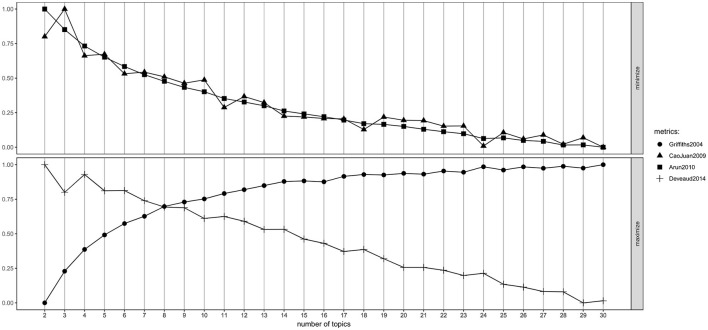
Line plot representing the performances of four different methods for estimating the most accurate number of topics in the corpus of documents.

The number of topics has been selected using the “ldatuning” function. Anyway, three out of the four methods used to estimate the correct number of topics seem to have reached a plateau [minimum for the first two methods (32, 33) that aim at the minimisation of the differences internal to the set of topics; maximum for the third (34), that aims at the maximisation of the differences between topics] after the 20th topic (Figure 9). As in other reported examples (36), the fourth method (35) fails to agree with the rest, giving back non informative results (perfect maximisation at two topics). For the sake of brevity in the subsequent discussion, we decided to use 24 as a parameter to feed the LDA model and assigned labels to each of them. Twenty-four was, in fact, the minimal number of topics to satisfy the tendencies of the three models towards their minimum and maximum values.

The 24 topics extracted by the LDA model, and the relative 18 most appropriate terms for each topic, are reported graphically in the Supplementary material 1. The same topics are labelled *a posteriori* through a collaborative process of labelling, which is reported in Table 3 (only the labels) and extensively in Supplementary material 2. In some instances, the process of labelling required further reading of the abstracts included in the cluster since the words that emerged from the topic analysis were difficult to interpret without further context due to their generality.

**Table 3:** Labelling of the numbered topic extracted from the LDA model in relation to the 18 most appropriate words (N, number of the topic).

**N**.	**Words**	**Label**
1	owner, behaviour, group, differ, semen, success, human, gaze, pet, work, breed, blind, rate, use, communic, high, train, interact	TRAINING: HUMAN DOG INTERACTION
2	child, famili, parent, ASD, autism, behaviour, use, signific, interact, impact, social, benefit, find, effect, disord, measur, increas, aad	AUTISM SPECTRUM DISORDER (ASD)
3	behaviour, puppi, age, distract, assess, predict, train, trait, month, fear, measur, score, associ, reliabl, signific, week, high, respons	PUPPY TRAINING PREDICTION
4	veteran, benefit, theme, use, challeng, life, qualit, particip, sds, find, experi, public, PTSD, analysi, interview, relat, assist, explor	PTSD: BENEFITS
5	impair, visual, mobil, use, owner, vision, aid, particip, blind, social, experi, increas, life, access, health, user, find, activ	VISUAL IMPARIMENTS
6	PTSD, veteran, symptom, use, effect, particip, group, train, sleep, report, partner, signific, militari, intervent, treatment, follow, associ, qualiti	PTSD: SYMPTOMS
7	disabl, use, work, law, case, ethic, veterinari, paper, practic, blind, provid, articl, human, person, good, need, discuss, approach	REGULATIONS AND ETHICS
8	alert, dad, owner, train, hip, rang, perform, glucos, behaviour, sensit, high, posit, signific, retriev, diabet, month, use, age	DIABETES ALERT DOGS (DAD)
9	hear, health, life, social, individu, particip, qualiti, wellb, physic, impact, group, function, signific, caregiv, relat, receiv, emot, effect	HEARING IMPAIRMENTS
10	handler, disabl, owner, relationship, experi, individu, social, benefit, provid, person, use, time, first, life, includ, increas, train, human	HANDLER-DOG RELATIONSHIP
11	facil, accredit, behaviour, place, non, cryopreserv, human, towards, disabl, train, among, state, use, canin, role, embryo, cultur, differ	BREEDING MANAGEMENT
12	behaviour, test, use, score, breed, work, differ, high, train, relat, owner, select, factor, show, tempera, person, fear, find	BEHAVIOURAL TESTING
13	train, health, particip, disabl, sdtp, work, therapi, categori, staff, care, mental, knowledg, use, polici, low, profession, month	TRAINING PROCEDURES: KNOWLEDGE AND POLICIES
14	age, breed, test, use, retriev, risk, neuter, litter, time, labrador, increas, factor, later, associ, diseas, month, femal, pup	LABRADOR RETREIVER BREED
15	mobil, success, train, measur, use, cognit, work, predict, devic, task, outcom, travel, function, ambul, differ, suggest, test, provid	MOBILITY (AND GUIDE) DOGS PRACTICALITIES
16	train, use, week, frequenc, student, assist, session, group, patient, interfer, report, human, affect, cough, time, sever, provid, task	TRAINING ORGANISATION
17	behaviour, social, famili, increas, train, child, interact, program, use, find, human, subject, task, person, woman, inmat, provid, respons	ASSISTANCE DOGS IN FAMILY AND SOCIETY
18	puppi, behaviour, raiser, train, rais, program, success, social, use, questionnair, factor, month, volunt, effect, time, develop, practic, organ	PUPPY RAISERS
19	occup, use, public, type, work, attack, experi, welfar, therapi, therapist, datum, particip, survey, assess, challeng, inform, report, provid	CHALLENGES, KNOWLEDGE AND ATTITUDES TOWARDS ASSISTANCE DOGS
20	patient, diabet, care, use, cost, campaign, blind, expens, provid, benefit, law, financi, medic, type, includ, arous, life, disabl	DIABETES POLICIES
21	wheelchair, user, signific, manual, mobil, use, time, test, diet, admob, train, perform, without, particip, msd, effect, improv, high	MOBILITY IMPAIRMENT
22	har, use, polici, walk, handl, visual, state, sign, sleep, work, type, show, right, individu, among, signific, collar, communic	GUIDE DOG HANDLING
23	seizur, alert, sampl, detect, scent, train, stress, level, human, epilepsi, canin, sweat, patient, serum, use, owner, two, pet	SEIZURE ALERT DOGS
24	cortisol, behaviour, program, associ, compar, cbarq, train, releas, differ, level, questionnair, companion, one, use, control, user, outcom, min	DOG BEHAVIOUR, PERSONALITY AND WELFARE TESTING

## 4 Discussion

The current review allowed for the analysis of the literature on a growing sector using innovative techniques of textual analysis that could integrate the work done with traditional reviewing methods. Text mining and topic analysis methods could also help reveal subtle emerging trends that are not the primary focus of the scientific articles in which they are mentioned. Moreover, the analysis of existing literature could evidence important gaps in our knowledge of the field and orient future research.

In terms of the publication characteristics of the included articles, the analysis of publication dates provides evidence of a growing interest in this subject, as evidenced by the fact that the number of articles published in the last 5-year period is twice that of the previous 5-year period. This trend may be partly attributed to the exponential growth in global scientific production that occurs on an annual basis (37). Nevertheless, it also reflects an increasing focus on the topic.

Moreover, the descriptive analysis of scientific journals publishing articles on the subject reveals that those in the field of veterinary and animal science are the most prolific. Nevertheless, this does not necessarily imply that the subject matter is limited to veterinary or agricultural/biological science disciplines. In fact, it should be noted that the articles are distributed across a wide range of journals belonging to many disciplinary fields. This may simply indicate a wider distribution across several journals and subjects in other (non-veterinary) fields. This is corroborated by the analysis of disciplinary areas, which reveals that the field of human health and social sciences is also well-represented. Indeed, while the areas of veterinary medicine and agricultural and biological sciences account for ~35% of the total, over 40% of the areas of interest fall within the disciplines of medicine (which mainly refers to humans), social sciences, psychology, health professions, and nursing. The topic analysis shown in the results confirms the multi- and interdisciplinary character of the topic, as will be further discussed in the following paragraphs. An interdisciplinary approach was also selected *a priori* for this review, in accordance with the approach previously proposed by Pierce and Dreschel (11). This was done in order to optimise the potential of the analytical techniques employed (text mining and topic analysis) and to permit the emergence of topics without subject area constraints. Finally, as predictable, slightly < 70% of the scientific production on the topic comes from the English-speaking countries that are at the top of the productive rates in virtually all the scientific publications, such as the USA, UK, Australia, and Canada. The geographic origin, tightly linked to the language selected but also to cultural and economic aspects of society, heavily influences the research process in determining the importance of some issues and the way to address them at both a practical and theoretical level.

### 4.1 Text mining

With regard to the initial step of text mining, the identification of the most relevant words raises some interesting considerations. Among the five most relevant terms associated with higher TF_IDF values, two (“veteran” and “PTSD”) were specifically related to contexts involving the rehabilitation of army veterans from conditions of PTSD. This indicates the weight of the above-mentioned countries [especially the USA (38)], all of which possess an active army and a huge number of army veterans, in determining the centrality of this topic. The World Veteran Federation (WVF), which represents more than 60 million army veterans (almost half, 30 million, living in the USA and UK), advocated for a more holistic approach to caring for veterans (39). Assistance dogs are one of the measures adopted to counteract the elevated rate of suicides (40, 41) (in some of the so-called “western countries”) associated with conditions of psychological suffering (42). These two words were highly correlated to each other, and the term “PTSD” was highly correlated to a range of words describing psychological suffering and the dramatic effects associated with it. A rather standalone term in the correlation table was “waitlist,” possibly indicating that despite the halving in the number of army veterans in the USA during the last 30 years, the demand for this type of support exceeds the supply possibilities, leading to potentially dramatic effects (43, 44). The extreme relevance of these words in our collection of documents indicates both a great specificity of a topic that uses peculiar definitions and a great diffusion in terms of scientific prolificacy, aimed at improving this service important to several societies (45).

Most of the remaining relevant words refer to generic terms used across different fields and activities, such as “behaviour,” “owner,” “train,” “test,” “participant,” “work,” “disabil,” and “handler.” The generality of these terms is associated with the lack of a high degree of correlation with other terms, indicating that these words did not appear in strong coincidence with other specific terms.

Another relevant term, “puppi,” indicates a semantic nucleus related to the precocious training process necessary for the development of the delicate skills of assistance dogs. As will be discussed later in the topic analysis, raising a dog to be an adequate companion for people suffering from psychophysical conditions is difficult. The general emotional skills that permeate the relationship with humans (and other animals) stem from a correct early socialisation process (46). At the same time, learning the cognitively demanding task that some assistance dogs have to perform is easier when the puppy education program starts at a young age (47). For these reasons, the attention given to the early stages of the dogs' life is high, resulting in the centrality of the term “puppi.” This is the only word from the ranking that refers to dogs, and the words correlating to it describe the difficulties of the raiser and the practical aspects of the training. However, no terms are evident that relate to potential canine distress.

The relevant terms “alert” and “seizure” are also highly correlated to one another, with both referring to assistance to individuals suffering from diabetes or other medical conditions that can result in crises and seizures. This field is one in which the ability of properly trained dogs to detect and alert to such crises is being tested (48–50). The term “Famili” correlates with the word “autism,” as well as with the terms indicating the various components of the family. This probably indicates that the entire family unit of individuals with autism may be involved in aspects related to the management of assistance dogs and their effects on the familial context as a whole (51).

### 4.2 Topic analysis

The most relevant terms are representative hints of the subdivision carried out by the LDA method. The 24 topics identified and summarised in Table 3 encompass a wide range of categories related to disabilities and the corresponding assistance dogs. Besides the centrality of the discussion about peculiar aspects of each specific disability, the collection of topics includes various tests and experiments conducted on both dogs and handlers, as well as dog training methods. An important theme that emerged is the relationship between assistance dogs and the person suffering from a disability, as well as with the puppy raisers and the family of the person with disability. Additionally, there are topics addressing legislation, ethics, and public perceptions of assistance dogs, along with a final cluster focused on dog breeding. It is worth noticing that only few topics focus on the dog instead of the human companion, and that the themes of dog behaviour and welfare are quite marginal in the whole collection. This trend is probably originated by past conceptions of the dog as a tool for assistance, while in the last years a general rise of awareness led to considering the importance of ensuring adequate care and attention for the welfare of the dog.

In fact, the dog's perspective seems to be underrepresented in the topics that address the diverse aspects of specific disabilities, which represent the majority of the outcomes of the analysis. The theme of the various users with disabilities that can benefit from assistance dogs (52) is obviously central in a literature analysis on assistance dogs because it constitutes the *raison d'être* of this sector. While each article mentions the figure of a peculiar assistance dog, the role of the dog is not always strictly the central focus of the articles. The articles deal with the conditions in their totality, sometimes lingering more extensively on the symptoms of a condition, or on the social consequences of it, and mentioning the dog only peripherally. Nevertheless, each condition requires the dogs to possess a specific set of complex cognitive and emotional abilities that must be nurtured by education, relationship, and training in order to properly work. For example, three of the 24 topics identified deal with medical alert dogs, specifically diabetes alert dogs and seizure alert dogs, seizures being possible symptoms of both conditions (diabetes and epilepsy) (53). In both categories emerged the word “training,” which is conducted differently between the two types of medical alert dogs: seizure alert dogs' training focuses on responding to subtle human behavioural cues (54), while diabetes alert dogs work on odour detection, specifically glycaemia level fluctuations identified in saliva samples during the training sessions (55). For both trainings, it is recommended to anticipate the person's crisis using a reward-based operant conditioning paradigm, to guarantee a good level of welfare in dogs. There is in fact evidence that using only reward-based (positive reinforcement) training techniques is more effective than aversive, punishment-based methods, or a combination of both approaches (56, 57).

Another aspect that has emerged from the topics identified is more related to the treatment of these diseases, in more general terms of care policies and the quality of life of patients. Moreover, in addition to the effective assistance provided by assistance dogs, it is important to bear in mind that any training, pairing and employment of dogs is subject to a cost-benefit rule.

Other two topics emerged from the text mining dealt with guide dogs for blind people, and both highlighted their primary role as mobility aids. Individuals who are blind, especially those who have recently lost their sight, face significant challenges when navigating crowded areas or unfamiliar environments and can experience social isolation, increased reliance on others, depression, and a marked decline in their quality of life (58). These dogs help increase mobility by navigating around obstacles, locating destinations, and supporting the independence of their handlers. To achieve this, guide dogs must wear special harnesses that their handlers can hold, and some guide dog handlers suggest that navigating with a dog is less exhausting than using the long white cane because it requires less effort (59). Given this, it is likely that the topics (and relative articles) associated with guide and mobility dogs are not entirely distinct in topic analysis. In fact, mobility dogs assist people with physical disabilities that limit their motor functions. These dogs are engaged in everyday life to perform tasks such as opening and closing doors, carrying mobile phones or other objects, switching lights on and off, picking up objects that have fallen to the floor, loading washing machine baskets, calling for help from other people on command, pressing the remote assistance button, help in dressing and undressing, pressing the pedestrian crossing button, etc. (60).

As previously mentioned, a topic that emerges with a high degree of definiteness from the analysis of relevant words is related to PTSD. Some of the tasks performed by PTSD assistance dogs include providing support during moments of anxiety and reminding their owners to take their medication (43, 61). Most of the tasks of these dogs are triggered by the context rather than by voluntary instructions from the handler, as the person may not be able to request the animal's help. Therefore, it is essential that the dog is able to recognise symptoms early and intervene independently to alleviate the impact of the illness on the handler (3). Context-dependent emergency situations may include a PTSD dog waking the affected person during a nightmare, and it's interesting to notice that “sleep” is one of the words that emerged from the topic “PTSD symptoms,” as nightmares and disrupted sleep patterns are common symptoms of PTSD (62). Another task these dogs may perform, as reported in the Rodriguez et al. (63) study, is the “cover or watch my back” task. This activity helps to reduce PTSD symptoms of hypervigilance and is believed to replicate aspects of military camaraderie, where soldiers guard each other's blind spots during combat (63). These dogs are extensively employed to assist army veterans, as discussed earlier in the discussion section.

A hearing dog is a type of assistance dog trained to alert their deaf owner to several specific sounds by performing a range of alerting behaviours. These behaviours may include touching the owner's foot with a paw, nudging them with their nose, sitting in front of the deaf individual and bringing the person towards the source of the noise, or lying down in the case of a fire alarm. The dogs are trained to recognise different environmental sounds, both at home and outside, including fire and smoke alarms, kitchen timers, doorbells, telephones, babies crying, calling of owner's name, or other keywords (64). The other relevant words of this topic pertain to the social and emotional realms, such as “social,” “quality,” “wellbeing,” and “emotion.” This is likely due to the fact that the Deaf community often experiences a sense of isolation from society (65).

Finally, Autism Spectrum Disorder (ASD) is a condition often characterised by limited social skills, with individuals having difficulties understanding social cues and communicating with others. The main role of ASD assistance dogs is to facilitate social interactions between individuals with ASD and others, acting as mediators in these situations (66). The dog's actions are often mediated by the parent, and the central role of the family and parents in relation to the child suffering from ASD emerged in the first three most relevant words from the text mining (67). The child is generally encouraged to walk alongside the dog, which provides the child with a point of reference and security in relation to the context. In fact, the dog can help children, but also adults suffering from this disorder, modify their behaviours by introducing a routine, interrupting repetitive behaviours and helping to cope with unfamiliar environments. In addition, these dogs can learn to lie on the person when the person is extremely distressed, providing a tactile stimulus that helps to improve the person's state of mind by making them feel calmer and safer (68, 69).

A second group of topics gathers together themes relating to the several aspects involved in preparing the dogs for their role. This process requires a great investment in terms of time and money. Considering the limited availability of resources, many efforts are aimed at optimising the selection and training of the best subjects. This optimization is possible only through a precise evaluation of the characteristics that assistance dogs must possess (70–72). Frequently, the chosen puppies, upon reaching an adequate age, undergo training sessions tailored according to the type of disability the person they are assigned to assist is suffering from. The training protocols are more likely to vary between training centres rather than following shared standards and evidence-based information and techniques (73). Nevertheless, there is widespread consensus on adopting methods that exclude the use of coercive actions, basing the dog's training instead on positive reinforcement, as briefly mentioned above in the “medical alert dogs” paragraph. This approach involves increasing the frequency of desired behaviours through rewards, while any undesired behaviour is ignored, without resorting to coercion of any kind. This methodology aims to develop a positive relationship between the dog and the assisted person, enhancing the dog's natural aptitudes and safeguarding his/her welfare (74, 75).

This training process requires the devotion of a great deal of time and resources, so several items emerged from the topic analysis concerning the development of tests with potential predictive value for dog training outcomes (76–78). These tests could be performed on puppies (before they reach 3 months of age) to select the most suitable candidates for assistance dog roles. Despite the influence of early life learning on adult behaviour, it is generally accepted that administering tests to puppies may be unreliable for predicting a dog's future behaviour, with only a few studies demonstrating a correlation between puppy test results and the training outcomes of working dogs (79–81). Behavioural testing is also employed at the end of the dog's training path, where a final test is performed to evaluate the preparation and readiness of dogs to be entrusted to individuals suffering from a disability (70). The assessment of dogs' behavioural attitudes and personalities extends beyond the use of test batteries to include questionnaires (such as the C-BARQ) (82) and the collection of biological samples to study the physiological responses of dogs involved in assistance activities (e.g., cortisol levels as indicators of stress) (83). In fact, stressful situations and environments can jeopardise the animal's wellbeing by negatively modulating the subject's neurohormonal balance. The ability to manage one's emotions and emotional stress is essential for selected individuals (84). Several factors may predispose the animal to an insufficient state of wellbeing, including owners' lack of knowledge about dogs' needs and necessary care, high and not always subsidised costs, expectations about the tasks the dog will perform, and inadequate matching of the animal's needs with those of the owner and family (85). Training procedures do not solely concentrate on the dog; they also encompass the person with disability who is assisted by a dog. At a certain point in the training process, the dyad (or the couple formed by pairing the dog and the assisted individual), becomes the primary focus of training. What commonly happens is that specialised training centres prepare dog litter to assist people with one specific disability. Once the dogs are grown enough and have passed specific attitudinal and performance trials, they are claimed “assistance dogs” by the assistance dog service provider (and depending on the country, they can obtain public recognition). In a second phase, these dogs are matched to the person who needs assistance, and a follow-up period may occur during which the dyad is supported by a dog trainer to assess the optimal integration of the dog in the new family environment (86). Another possibility is that an already established dyad starts a specific learning path tailored on them and their needs, applying a cognitive-relational approach led by a dog trainer (87). In both scenarios, after a settling-in period, the mechanics of applying the learned notions to the dogs should be integrated with an emotional motivation that fosters a sense of fulfilment in the dog through its closeness to the assisted person. To test the dyad dynamic, some words of the first topics suggest that behavioural observations can be taken into account, for instance studying the dog's gazing behaviour towards its owner (88, 89).

It is possible to address a further set of topics within a relational sphere framework. Considering that one prerequisite to define an assistance dog is that the dog is directly cared for by the patient in an ongoing one-to-one relationship or by another caregiver, such as a parent or spouse, it is not surprising that one macro area refers to the relational sphere. Dogs are animals that share with humans a great part of their most recent evolutionary history, making these two species particularly tuned to their respective emotional expressions. The fundamental element ensuring long-lasting cooperation, positive for both members of the couple, is a good relationship (90). Assistance dogs play a crucial role in enabling people with disabilities to achieve an optimal level of functional independence in daily activities, thus helping to improve social participation and reducing the need to seek external help. Furthermore, owing to the profound bond many humans share with dogs, assistance dogs often serve as sources of support in various ways, allowing their handlers or owners to gain emotional and psychological benefits (91, 92).

Moreover, individuals with disabilities frequently rely on familiar support. Consequently, this responsibility may have a direct or indirect impact on the health, wellbeing, and daily life management of the entire family system (93). In some cases, the impact of the introduction of an assistance dog extends to family members, indirectly reducing the level of anxiety and making them feel safer in dealing with the disability of their relative (94). This social network in turn establishes a relationship with the dog and supports the person suffering from the disability in the optimal care of the animal.

Another key figure, who forms a close bond with the dog and provides care, is the puppy raiser or puppy walker. These individuals, often volunteers from outside breeding and training facilities, foster prospective working dogs after weaning, until they are ready for early and advanced training (95). Puppy walkers are essential for most recognised assistance dog organisations as the puppy's experience with his/her raiser is crucial for behavioural development (96, 97). Puppy raisers teach basic obedience and socialisation skills, introducing new stimuli to reduce future fear-based responses. During this path, they are supported and receive guidance from the host organisation to prevent undesirable behaviours through proper puppy education programmes (98).

Expanding beyond the individuals who directly take care of assistance dogs, it is essential to consider that the dog interacts (and relates) with human society at large, and therefore with beliefs, perceptions, attitudes, customs, and habits. Increased awareness of the needs of individuals suffering from disability and their assistance dogs can drive collective change, leading to enhanced rights, accessibility policies, and more comprehensive industry regulations and standards, affecting the lives of both the owners and the dogs (99, 100).

Two final sets of topics address specific aspects of assistance dogs: “knowledge and context” and breeding.

With respect to the former, we draw upon the work of Pierce and Dreschel (11), which similarly encompasses articles pertaining to legislation, ethics, and the challenges faced by individuals with disabilities and their assistance dogs. As already mentioned, nowadays, significant bias still exists in public perception regarding people with disabilities. These negative attitudes disempower them and lead to their social exclusion and isolation (101). This sense of marginalisation can be exacerbated by the presence of a service dog, as it may attract negative attention or judgment from the public, as reported by 20% of participants with a service dog in the study by Rodriguez et al. (102). The same research identified additional challenges in owning an assistance dog, including caring for the dog and his/her health needs, dog behavioural issues, lifestyle changes to address dog needs, the lack of public education and access difficulties (102). At both European and global levels, there is a lack of comprehensive legislation addressing the fundamental issues concerning assistance dogs for individuals with disabilities. Currently, no unambiguous and universally accepted definition of “assistance dog for people with disabilities” has been established through a binding legal act. This uncertainty leads to accessibility issues, as “assistance dogs” are not properly recognised. Additionally, there is a lack of precise education and training programmes to ensure that assistance dogs acquire skills far beyond those of pet dogs, and the certification system remains fragmented or non-existent in some countries. Assistance dogs are a form of sentient aid to which persons with disabilities are entitled under the December 2006 “United Nations Convention on the Rights of Persons with Disabilities,” which specifically addresses accessibility rights (103).

Finally, it is evident that factors such as breed, genetics, and training programmes significantly influence the success probability of trainee dogs (70). Therefore, the role of the breeder is crucial, as they must care for the mothers during selection, mating, pregnancy, and parturition, as well as attend to the puppy litter during the weaning period. In addition, the accurate selection of genetic traits that are considered favourable, unfavourable, or to be avoided (e.g., predisposition to genetic diseases) is essential for the reproduction process (71, 104, 105). Breeders can be independent individuals that provide dogs to the assistance dogs training centres that require it, but many assistance dog organisations maintain their own breeding programs (106). Assistance dog facilities typically utilise a limited number of breeds in their programmes, primarily Labrador Retrievers, Golden Retrievers, and their mixes, as emerged also from the text mining. Some facilities also use German Shepherds (107), Labradoodles, and Goldendoodles (108). Various studies on Labrador Retrievers have been conducted, and one of them found Labradors to be the breed most likely to reach retirement, meaning they successfully complete their training, are matched, and work as guide dogs until retirement (109). They exhibit higher nerve stability, greater cooperation attitude than German Shepherds, and are the least fearful among the breeds assessed (110). Another study, comparing four dog breeds for guide work, found Labrador Retrievers scored higher in traits such as courage, resilience, and friendliness (107). Additionally, research on the behaviour of dog breeds indicated that Labradors are globally ranked highly for low aggression levels and score higher in trainability (111).

The topic of Labrador Retriever breed is an example of a transversal topic that is likely to emerge more easily through the application of text mining and topic analysis than through classical review methods. This is because LDA allows the identification of semantic structures and relationships hidden in the text (112, 113). This methodological aspect, along with the disparity in the date of the search and the inclusion criteria between the articles, may account for the slight discrepancies between the topics identified in this review and those identified by Pierce and Dreschel (11). The authors identified the previously mentioned “knowledge and context” category, a category on “health and management of service dogs” (including aspects on their breeding, selection, training, evaluation, and health), and an “other topics” category, including handler/team elements, a focus on specific SD types, and other aspects. In general, it can be observed that many of the topics identified are similar, and in some cases, they are simply categorised or conceptualised in a slightly different manner. Nevertheless, as a limitation of the current study, it is noteworthy that from the current analysis the “health” issues of service dogs did not completely emerge, despite the presence of articles on case reports of various pathologies in assistance dogs and issues related to reproductive and orthopaedic diseases. This may be attributed to the presence in the articles of a diverse array of veterinary terminology that is peculiar to the field, which constrains the capacity for clustering the words.

**Figure 10:**
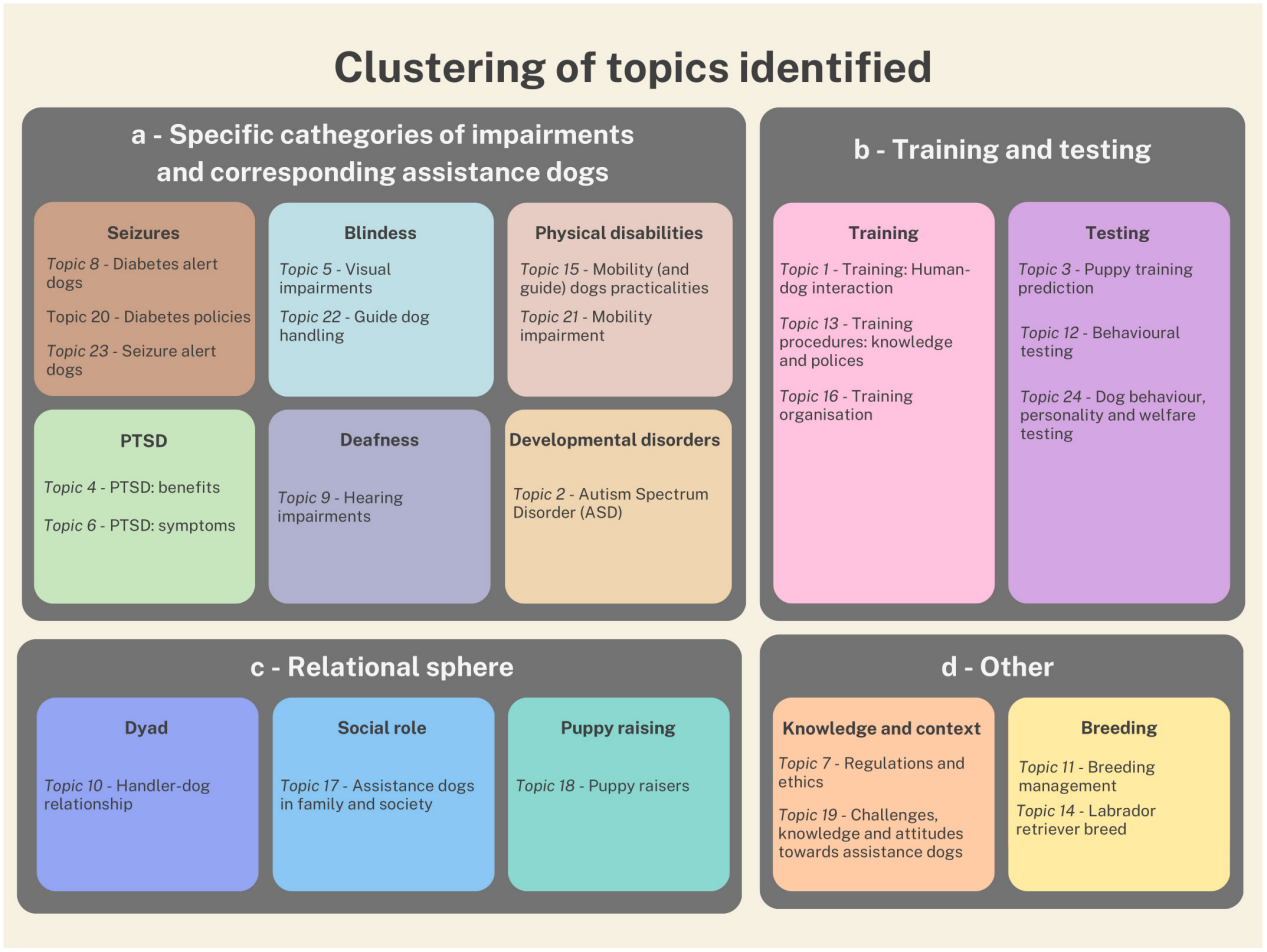
Topics emerged and categories identified.

For the purpose of providing an overview of the main themes addressed in this section, Figure 10 offers a conceptual framework for organising them into four macro-categories: specific disabilities (and corresponding assistance dogs); training and testing; the relational sphere and other (knowledge and context, breeding).

## 5 Conclusion

The use of automatic computation tools is an unstoppable process that is taking place in every aspect of our society, including scientific research. Its fast-paced progress makes it a precious tool to assist, speed up, and perfect the work of humans involving information synthesis and analysis. We decided to experiment with text mining and topic analysis techniques to survey the literature regarding assistance dogs. While these techniques are designed to perform the analysis of overly vast collections of documents that would be unpractical to tackle manually, our “limited” corpus of 450 abstracts seemed big enough to test these information-crunching techniques, which necessitate a great amount of data to work properly, and at the same time manage to supervise it manually. This number is thus appropriate for our aim, which was not to have a fully automatized and trustworthy review of a completely unexplored subject, but to test the power of these tools in assisting a reviewing activity on a topic familiar to us and the scientific community. The words and topics identified permitted us to make considerations and link concepts (some of which were readily intuitive and others less so) related to assistance dogs in order to obtain an understanding of the state of knowledge in the literature.

From an epistemological standpoint, the objective is not to implement an exhaustive automated analysis of the literary corpus; rather, it is essential to recognise that the labelling and categorisation process inherently requires an intermediary and interpretive role for authors. Nevertheless, text-mining and topic analysis techniques guaranteed a surprising degree of speed and precision. In this way, the conceptual discussion of the various themes can be backed-up by solid, quantitative data regarding the constituent elements of the discourse.
