# Supplementary material for: An overview of the literature on assistance dogs using text mining and topic analysis
Source: Front Vet Sci. 2024 Dec 11;11:1463332. doi: 10.3389/fvets.2024.1463332 (PMC11669006; doi:10.3389/fvets.2024.1463332)
Supplement: Supplementary file 1 [file Table_1.docx]

Supplementary Material


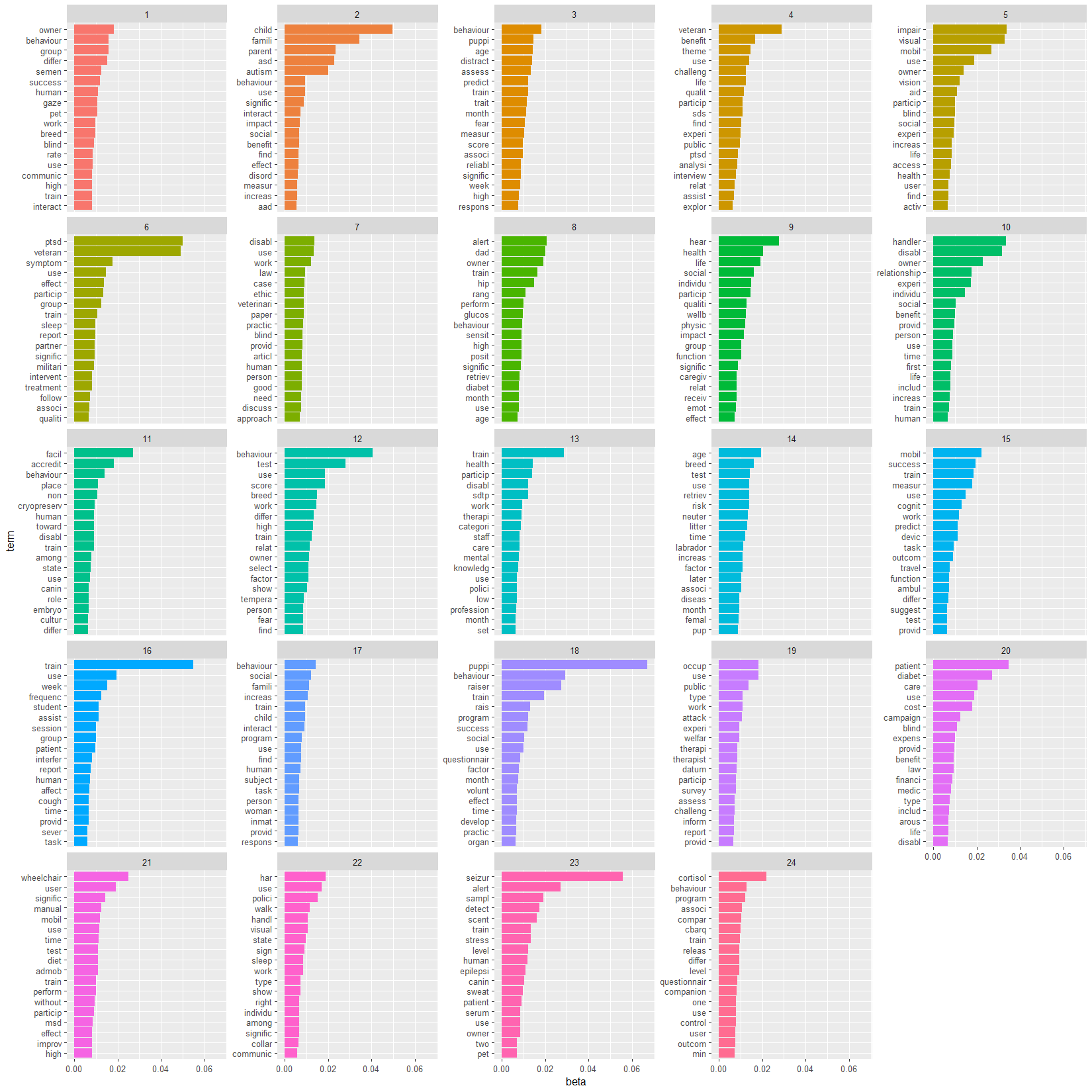


**Supplementary Figure 1**: Bar plots illustrating the 18 most appropriate terms for each of the 24 topics extracted by the LDA model. The beta value on the x axis indicated the relative probability of each term belonging to that topic.

**Supplementary Table 1**: Process of labelling with explanation

| N. | Words | Label | Explanation |
| --- | --- | --- | --- |
| 1 | owner, behaviour, group, differ, semen, success, human, gaze, pet, work, breed, blind, rate, use, communic, high, train, interact | TRAINING: HUMAN-DOG INTERACTION | The initial topic can be attributed to the field of interspecific behaviour, communication, training and breeding aspects, which can be summarised as human-dog interaction. This topic is likely to include articles on scientific assessments of behaviour towards the owner in terms of interaction, communication and other cognitive/relational skills, which are also relevant in determining training success. |
| 2 | child, famil, parent, ASD, autism, behaviour, use, signific, interact, impact, social, benefit, find, effect, disord, measur, increas, aad | AUTISM SPECTRUM DISORDER (ASD) | The second topic’s most relevant words refer to the domain of young population suffering from autism spectrum disorder, that could benefit from the presence of an assistance dog. The focus is not only on the individual suffering from the condition, but on the whole social context of the family. |
| 3 | behaviour, puppi, age, distract, assess, predict, train, trait, month, fear, measur, score, associ, reliabl, signific, week, high, respons | PUPPY TRAINING PREDICTION | The third topic focuses on the delicate training process of the puppies that will be involved in assistance activities and on some indicators that could be useful predictors of future performances. The top-ranking terms in this topic are some of the most relevant ones emerged from the text mining analysis. |
| 4 | veteran, benefit, theme, use, challeng, life, qualit, particip, sds, find, experi, public, ptsd, analysi, interview, relat, assist, explor | PTSD: BENEFITS | Other top-ranking terms in the list of most relevant words (TFIDF) are veteran and ptsd, the nucleus of the fourth topic, that describes the benefits associated to the presence of assistance dogs. |
| 5 | impair, visual, mobil, use, owner, vision, aid, particip, blind, social, experi, increas, life, access, health, user, find, activ | VISUAL IMPARIMENTS | The fifth topic concerns the distinctive population of visually impaired individuals who benefit from the assistance of a trained dog to facilitate their mobility, improve their independence in daily activities and enhance their overall quality of life. |
| 6 | ptsd, veteran, symptom, use, effect, particip, group, train, sleep, report, partner, signific, militari, intervent, treatment, follow, associ, qualiti | PTSD: SYMPTOMS | As in topic 4, once again, some of the most relevant terms (“veteran” and “ptsd”) emerge as nuclei of a topic, this time declined on the cause and symptoms of their conditions that are probably often described in the articles. |
| 7 | disabl, use, work, law, case, ethic, veterinari, paper, practic, blind, provid, articl, human, person, good, need, discuss, approach | REGULATIONS AND ETHICS | The three most characterizing term of this topic are “disable”, “use(r)“and “work”. As explained by the rest of words, the topic explores the practical difficulties faced by people with disabilities in participating to public life that can be tackled by a change in society by new laws and a redefinition of the ethical assumptions that shape the perception of the world by the person with disability and vice versa. In particular, it mentions “blind”, this could be due to the major regulations and public recognition for guide dogs compared to other types of assistance dogs. |
| 8 | alert, dad, owner, train, hip, rang, perform, glucos, behaviour, sensit, high, posit, signific, retriev, diabet, month, use, age | DIABETES ALERT DOGS (DAD) | The eighth topic goes back to focusing on a special population of assistance dogs: alert dogs for diabetic patients. The other terms contained in the topic suggest the dynamic of this assistance process: the alert dogs detect hints of an upcoming hyper- or hypo-glycaemic crisis and provide to alert the user or other relatives. |
| 9 | hear, health, life, social, individu, particip, qualiti, wellb, physic, impact, group, function, signific, caregiv, relat, receiv, emot, effect | HEARING IMPAIRMENTS | In the ninth topic, a seemingly generic list of terms gains definition when read declined according to the most relevant word of the topic “hear”. This topic is likely to encompass studies referring to a specific category of assistance dogs that assist deaf people, namely hearing dogs. |
| 10 | handler, disabl, owner, relationship, experi, individu, social, benefit, provid, person, use, time, first, life, includ, increas, train, human | HANDLER-DOG RELATIONSHIP | The tenth topic concerns the theme of the relationship between dogs and handlers. Despite being a non-strictly technical aspect of the assistance dynamic, it is nonetheless a crucial element, a prerequisite, in determining the success and the wellbeing of the dyad throughout their life together. |
| 11 | facil, accredit, behaviour, place, non, cryopreserv, human, toward, disabl, train, among, state, use, canin, role, embryo, cultur, differ | BREEDING MANAGEMENT | This topic focuses on the breeding facilities and accreditations necessary to breed assistance dogs and evaluate them at the end of the training path. We could hypothesise that it involves consideration on the breeding techniques as suggested by the word “cryopreserv”, being cryopreservation, a technique used to preserve sperm, fertilized eggs and embryos (listed as "embryo"). |
| 12 | behaviour, test, use, score, breed, work, differ, high, train, relat, owner, select, factor, show, tempera, person, fear, find | BEHAVIOURAL TESTING | The focus of this topic is on behavioural tests that could be used to assess the predisposition of certain dogs to become assistance dogs. It also includes the final tests performed to evaluate the preparation and readiness of trained dogs to be entrusted to disabled individuals. |
| 13 | train, health, particip, disabl, sdtp, work, therapi, categori, staff, care, mental, knowledg, use, polici, low, profession, month | TRAINING PROCEDURES: KNOWLEDGE AND POLICIES | This topic principally concerns the training of canines; however, it appears to encompass this subject matter more from a sociological vantage point, delving into the domain of knowledge and policies pertaining to the procedures associated with canine training. |
| 14 | age, breed, test, use, retriev, risk, neuter, litter, time, labrador, increas, factor, later, associ, diseas, month, femal, pup | LABRADOR RETREIVER BREED | The fourteenth topic groups the interest in special breeds of dogs that are notoriously docile, prone to human contact, such as retrievers (Labrador). |
| 15 | mobil, success, train, measur, use, cognit, work, predict, devic, task, outcom, travel, function, ambul, differ, suggest, test, provid | MOBILITY (AND GUIDE) DOGS PRACTICALITIES | Topic fifteen groups several interconnected elements regarding features of guide and mobility dogs that, despite being dedicated to populations suffering from different disabilities, share similar tasks in improving or even allowing the possibility of movement in the world for this people. This extremely delicate and complicated task necessitates a long, complex, and costly training that would benefit from instruments of success prediction. |
| 16 | train, use, week, frequenc, student, assist, session, group, patient, interfer, report, human, affect, cough, time, sever, provid, task | TRAINING ORGANISATION | Topic sixteen focus on the dog training structure, focusing on the training organization in time, mentioning words like “week” and “frequenc” that could refer to the frequency of the weekly trainings, assistance dogs undergo. |
| 17 | behaviour, social, famili, increas, train, child, interact, program, use, find, human, subject, task, person, woman, inmat, provid, respons | ASSISTANCE DOGS IN FAMILY AND SOCIETY | The words within this topic appear to emphasise the social aspects of living with an assistance dog, as evidenced by the term "interact" which suggests the relationship between the dog and various social entities, including, for example "family", "child" and "woman". |
| 18 | puppi, behaviour, raiser, train, rais, program, success, social, use, questionnair, factor, month, volunt, effect, time, develop, practic, organ | PUPPY RAISERS | Puppy raisers are figures which play a significant role in shaping the puppy behaviour and performing the first steps of training. Their socialization program is a primary source of variation in the development of the canine profile and will influence the success of the assistance dog training. |
| 19 | occup, use, public, type, work, attack, experi, welfar, therapi, therapist, datum, particip, survey, assess, challeng, inform, report, provid | CHALLENGES, KNOWLEDGE AND ATTITUDES TOWARDS ASSISTANCE DOGS | This topic appears to examine the challenges associated with the employment of assistance dogs, both at the individual level (e.g. welfare, attack, etc.) and at the public level (appropriate information, survey). The initial word "occup" is likely linked to the words "therapi" and "therapist”, being occupational therapy a field that is often intertwined with assistance dogs. |
| 20 | patient, diabet, care, use, cost, campaign, blind, expens, provid, benefit, law, financi, medic, type, includ, arous, life, disabl | DIABETES POLICIES | The twentieth topic deals with the population suffering from diabetes and other related conditions that are greatly invalidating and would benefit from specialized policies to be dealt with. |
| 21 | wheelchair, user, signific, manual, mobil, use, time, test, diet, admob, train, perform, without, particip, msd, effect, improv, high | MOBILITY IMPAIRMENT | The 21st topic’s list of terms describes patients with impaired mobility (especially wheelchairs users), another major population of assistance dogs users. |
| 22 | har, use, polici, walk, handl, visual, state, sign, sleep, work, type, show, right, individu, among, signific, collar, communic | GUIDE DOG HANDLING | This topic describes the dog from a more utilitaristic perspective as “walking aid”. This is highlighted by the first word “harness” and by the following “use”, “walk”, but also “handling”. Since it specifies “visual”, we hypothesise that it deals with guide dog for the blind when moving in urban environments. |
| 23 | seizur, alert, sampl, detect, scent, train, stress, level, human, epilepsi, canin, sweat, patient, serum, use, owner, two, pet | SEIZURE ALERT DOGS | The focus is on alert dogs, but differently from topic 8, which focused on diabetes alert dogs, the alert is directed towards seizures caused by epilepsy and not by diabetes. |
| 24 | cortisol, behaviour, program, associ, compar, cbarq, train, releas, differ, level, questionnair, companion, one, use, control, user, outcom, min | DOG BEHAVIOUR, PERSONALITY AND WELFARE TESTING | Similarly to topic number 12, the final category addresses the assessment of dogs' behavioural attitudes and personality through the use of questionnaires (such as the C-BARQ). However, as indicated by the term "cortisol" it also involves studying the physiological responses of dogs participating in assistance activities by collecting biological samples (e.g., cortisol levels) to measure indicators of stress. These stress-studies allow making considerations on assistance dogs’ welfare. |
